# Different stages of emotional prosody processing in healthy ageing–evidence from behavioural responses, ERPs, tDCS, and tRNS

**DOI:** 10.1371/journal.pone.0270934

**Published:** 2022-07-21

**Authors:** Constantina Maltezou-Papastylianou, Riccardo Russo, Denise Wallace, Chelsea Harmsworth, Silke Paulmann

**Affiliations:** 1 Department of Psychology and Centre for Brain Science, University of Essex, Colchester, United Kingdom; 2 Department of Brain and Behavioural Sciences, Universita’ di Pavia, Pavia, Italy; CNRS - Université d’Aix-Marseille, FRANCE

## Abstract

Past research suggests that the ability to recognise the emotional intent of a speaker decreases as a function of age. Yet, few studies have looked at the underlying cause for this effect in a systematic way. This paper builds on the view that emotional prosody perception is a multi-stage process and explores which step of the recognition processing line is impaired in healthy ageing using time-sensitive event-related brain potentials (ERPs). Results suggest that early processes linked to salience detection as reflected in the P200 component and initial build-up of emotional representation as linked to a subsequent negative ERP component are largely unaffected in healthy ageing. The two groups show, however, emotional prosody recognition differences: older participants recognise emotional intentions of speakers less well than younger participants do. These findings were followed up by two neuro-stimulation studies specifically targeting the inferior frontal cortex to test if recognition improves during active stimulation relative to sham. Overall, results suggests that neither tDCS nor high-frequency tRNS stimulation at 2mA for 30 minutes facilitates emotional prosody recognition rates in healthy older adults.

## 1. Introduction

Effective communication in humans relies on an individual’s ability to express their own emotional state and to perceive that of others. One of the many modes through which such emotional states may be assessed is through speech, which contains both a verbal (lexical-semantic) and non-verbal (prosody) component. It is known that compared to the visual system, the auditory system in humans and other mammals develops much earlier [1]; it is therefore not surprising that humans seem to develop the ability to distinguish emotional cues from speech early on as well. Despite the dependence on our auditory system throughout our lives, it has been observed that as we approach late adulthood (sixty-five years of age and above) [2, 3], our auditory and cognitive abilities in perceiving emotional expressions in speech and faces are negatively affected. While this may be partly attributed to hearing loss, which is more common in older adults, this phenomenon has also been observed in healthy older adults with no hearing problems, implying that the causes behind this apparent decline in perception ability may be more complex [4–6].

Emotional speech, as outlined above, can be separated into information from lexical-semantic cues and information from voice characteristics, both of which are complex in their own right. This paper focuses on the latter, and particularly on the perception of emotions as conveyed through prosody. The term prosody refers to the voice characteristics that offer supplementary meaning to verbal communication, e.g. the melody of speech (‘how’ things are said, as opposed to ‘what’ is said). For example, the utterance of the word ‘right’ could be interpreted as a question, sarcastic remark, or as a confirmatory word, depending on how prosodic cues are varied by the speaker. Vocal prosodic features include, but are not limited to the perception of pitch, stress, intonation, rhythm and timbre, which can be acoustically analysed by measuring the frequency, duration, or amplitude, amongst other measures [7]. It is well documented that emotions expressed through prosody can be well recognised and that accurate detection of emotional prosody forms an important part of social interactions [7–9]. Yet, a number of past studies indicate that the ability to recognise the emotional intent of a speaker seems to decline with increasing age (e.g. [8, 10, 11]).

### 1.1. Studies using behavioural methodologies

The study of emotional expression based on prosodic features has captured the interest of scientists from multiple disciplines for decades now, ranging from computing (e.g. [12, 13]), to neuroscience and cognitive psychology (e.g. [14, 15]), to social and developmental psychology, and psycholinguistics (e.g. [16–19]). Collectively, these studies reveal the complexity of emotional prosody perception. Studies that have explored this complex endeavour across the human lifespan seem to suggest that older age is associated with a decline in our ability to recognise emotional speech, especially when it reflects negative emotions [4, 8, 20, 21]. Two of the contributing factors to this decline seem to be related to cognitive adjustments caused by hormonal changes (e.g. [21–24]) and naturally occurring brain atrophy in older adults (e.g. [23]).

Using behavioural methodologies, Laukka et al. compared emotional vocal expressions and music performances to examine the effects of aging in respect to speech and music emotional recognition [20, 25]. Their findings support the hypothesis that neuroanatomic changes in older age affect how emotional speech is perceived. Specifically, they and others argue that the left and right hemispheres undergo changes at different times (i.e. “the right hemisphere may age faster than the left” [26–28]). In additional point put forward by the authors to explain their data patterns refers to the idea that aging leads to a shift of focus on more positive emotions and goals in life which might allow older adults to better regulate negative experiences and emotions when compared to younger adults [29, 30]. This is known as the “positivity effect", which potentially leads older adults to assign negative emotion labels less frequently. This shift towards increasingly labelling different emotions as “positive” should be visible in confusion matrices.

Paulmann et al. [8] examined whether there are any individual differences in emotional speech recognition by investigating differences between age and gender groups. Using a forced-choice task, participants had to assign pre-recorded sentences to one of seven emotional categories: anger, disgust, fear, happiness, neutral, pleasant surprise and sadness. Results revealed no significant differences for emotional speech recognition in terms of gender; however, a clear decline was observed in participants of older age. Results of a discriminant analysis, which was carried out to predict emotional category membership from acoustic properties of stimuli that were incorrectly labelled, suggest that some errors made could be predicted through acoustic cues. For example, young participants’ misclassifications were largely linked to mean pitch of stimuli while older participants’ misclassifications were predominantly explained through durational cues [8].

Kiss and Ennis [31] also explored how well younger and older adults can recognise emotional prosody. They showed that older participants performed significantly worse compared to younger ones. Similar to Paulmann et al. [8] no emotion-specific deficit was observed, which means that all emotional categories were difficult to recognise. Additional evidence that emotional speech recognition declines with age comes from Orbelo et al. [21] who looked at both emotional and attitudinal prosody. They report that younger participants outperform older participants. Finally, a meta-analysis of twenty-eight data-sets confirmed that older adults had trouble recognising basic emotions regardless of the sensory input (i.e. visual, auditory) [11]. In short, a growing body of evidence suggests that healthy aging impacts emotional prosody recognition.

### 1.2. Studies using event-related potentials (ERPs)

While many ERP studies on emotional prosody processing have been published reporting results from younger participants (typically student populations; e.g. [32–34]), only a few studies have looked at the effects of aging on emotional prosody using this methodology. This is surprising given clear evidence that different stages of emotional speech processing (e.g. early sensory processing, integration of lexical-semantic information with prosodic information, emotional re-appraisal) can be studied using this technique, c.f. [35].

For instance, Paulmann and Kotz [36] showed that early emotional salience detection can be linked to the P200 component. Specifically, the authors report that neutral prosody can be distinguished from emotional prosody in this component. Moreover, Schirmer et al. [33] report that emotional prosody modulates word processing in the N400 time window, while Pell et al. [37] link appraisal of emotional attributes to the late-positive component (LPC). Similar findings for the LPC were observed by Zora, Rudner and Magnusson [38] who employed an auditory oddball task for identifying intermittent target stimuli from irrelevant background stimuli. They reported effects in different time windows (300–350 ms; 350–400 ms; 820–870 ms), depending on the study design and the stimuli (e.g. words, pseudowords) that were used. Further support for processing stages following initial salience detection has also been reported by Paulmann, Ott and Kotz [39] who explored emotional speech perception at different processing stages; the “later” negativity (280–480 ms) which they report was linked to a build-up of an emotional representation based on information retrieved at the earlier salience detection stage.

Collectively, results suggest that different ERP components can be linked to different emotional prosody perception sub-processes including emotional salience detection (P200) and building-up of emotional representations of speakers’ mental states (starting from ~300 ms; e.g., N300), emotional meaning processing (N400 components), as well as more in-depth, thorough emotional appraisal processes (e.g., LPC, LPP, late negativities). Crucially, not every paradigm used in the past reveals all ERP components within the same experimental design. Rather, differences in task instructions, stimuli, and presentation modes seem to affect ERP responses (also see [40], for discussion of such effects on emotional responses). However, ERPs have proven useful to provide insights into these different processing stages and might therefore be particularly suited to outline which emotional processing sub-stages are affected in aging participants that lead to decreased recognition rates in behavioural studies.

ERPs have already been used to address this issue in the visual, rather than auditory domain. For example, in a study testing emotional perception of pictures, older adults (56–81 years old) demonstrated high variability in an LPP component elicited by the different emotions, compared to their younger counterparts (19–22 years old) [41]. Interestingly, there were no age-group differences in the P200 component, suggesting that emotional perception differences between young and older listeners may be more pronounced during later stages. Whether similar effects can be found for emotional speech as opposed to picture stimuli will be tested in the current study. Some support for this hypothesis comes from Ruffman et al. [11] who noted in their meta-analysis that the so-called later processing stages, i.e. processes that require participants to infer emotional meaning or explicitly label emotions, may be more prone to age effects because these processing steps are mediated more strongly by regions specifically affected by age-related neuro-anatomical changes. Specifically, brain atrophy is prominent in areas such as the frontal lobe, an area linked to reduced performance in executive functioning, e.g. attention, decision-making, memory [4, 5, 42].

### 1.3. Studies using neuro-stimulation techniques

In an attempt to find solutions that help overcome emotion perception impairments, recent attention has been focused on neuro-stimulation techniques. The idea is to bolster neuronal activity through the application of electrical current and thereby increase synaptic efficacy leading to improved task performance. Neurons are stimulated by inducing a weak, constant and non-intrusive electrical current. Transcranial direct current stimulation (tDCS), for example, either increases (anodal) or decreases (cathodal) the likelihood of action potentials in neurons by directly modulating their resting membrane potential [43], which may produce immediate but temporary changes in brain functions (e.g. [44, 45]). For instance, there is evidence that tDCS can enhance emotion recognition of facial expressions; Brennan et al. [46] have recently shown that anodal stimulation of the left dorsolateral prefrontal cortex can increase performance on a facial emotion recognition task in both healthy (mean_age_ = 30 years) and mildly depressed patients (mean_age_ = 37). While performance increase was mild (2% - 3%), results suggest that stimulating frontal cortex areas can be beneficial for emotion recognition processes. Similar results had already been reported by Nitsche et al. [47] who showed that emotional face identification ability was improved after repeatedly exciting the prefrontal cortex. Specifically, participants showed faster reaction times when distinguishing between a neutral and an emotional face on screen.

In addition to tDCS, some studies have applied transcranial random noise stimulation (tRNS). In contrast to the constant current used in tDCS, tRNS generates an oscillating current around a randomly generated noise stimulation with a set range of frequencies; higher frequencies (> 100 Hz) appear to enhance neuronal network activation [48]. Similar to tDCS approaches, evidence from tRNS has also shown changes in emotional facial recognition after stimulation. For example, Yang and Banissy [49] administered three tasks to healthy older participants (60+ years old) before and after tRNS. In two of the three tasks participants sorted angry facial expressions by strength (sort 6 faces, simultaneously presented, from ‘least like’ to ‘most like’ angry), likewise with happy facial expressions. In the third task, participants identified a target face amongst six alternative faces. Participants were randomly assigned to either high frequency tRNS (100–640 Hz) at a current intensity of 1 mA or sham (5 s active) to the inferior frontal cortex for 20 minutes. Ramp-up/ramp–down in both conditions was 15 s. Results revealed significantly improved performance for the anger perception task in the active condition relative to sham, but no statistically significant changes in performance were found in the happy perception or identity tasks. Interestingly, another study applying the same task on younger adults, reported that participants receiving tRNS outperformed those in the sham condition [50]. Yet, other studies applying tRNS on the prefrontal cortex found no significant difference between active and sham conditions for changes in state mood [51] or when evaluating emotional facial expressions [52].

Overall, research suggests that neuro-stimulation can impact emotional face recognition, though the age of participants, task demands, and stimulation duration and strength may influence findings. Of critical importance to the current study is that all reported studies used facial expressions as stimuli and results from emotional prosody research have not yet been reported.

Nonetheless, a study by Francois-Nienaber, Meltzer and Rudzicz [53] used emotional prosodic stimuli and tDCS to shed light on the on-going debate whether emotional prosody is mediated more strongly by right-hemispheric than by left-hemispheric structures. The authors hypothesised that when the left hemisphere is stimulated (and the right hemisphere’s neuronal activity inhibited), lexical content will be attended to more strongly. They tested their hypothesis with fourteen younger adults, using tDCS during the T-RES (Test of Rating of Emotions in Speech) speech emotion rating paradigm. Although their hypothesis was not confirmed, the authors reported some emotion-specific effects, one of which showed that the perception of happiness is not affected by tDCS, while anger and fear perception are susceptible to stimulation [53]. Thus, findings suggest that emotional prosody perception can be altered with tDCS and that it is worth investigating emotion-specific effects.

### 1.4. Motivation for the current investigation

Multi-step models of emotional speech perception (e.g. [35, 54–56]) argue that emotional speech perception involves a number of compulsory steps that can be summarised as follows: first, listeners need to rapidly extract a range of different acoustic cues from the speech input. Based on this early extraction and combination of cues, emotional significance for the listener is determined. Subsequently, emotional cues are processed in more depth to derive full emotional meaning of the input. Finally, social consequences have to be evaluated and responses need to be prepared accordingly. These steps have largely been confirmed in ERP studies (see summary above). Important for the current investigation is the assumption that any disruption in this processing sequence could have the potential of altering the ability to perceive the emotional intent of the speaker (reflected in recognition rates).

The present investigation is exploring which emotional prosody processing step is affected by healthy ageing to help shed light on why older adults are often outperformed by younger adults in emotional prosody recognition tasks. In Study 1, we examine two stages of emotional prosody perception by means of time-sensitive ERPs; specifically, the P200 ERP component, which has been linked to emotional salience detection [36, 57], and a later negativity previously linked to build-up of emotional representation (e.g., subsequently facilitating meaning processing) are of specific interest, e.g. [57]. We also collect accuracy responses. All dependent variables are compared between young adults and healthy ageing adults. In addition, following [8] we evaluate whether there are age-related patterns in the way certain emotions are misperceived from acoustic features, which in turn might affect individuals’ responses. If a decline of emotional prosody recognition in older adults is primarily linked to age-related neuroanatomical changes, we expect more severe differences between older and young listeners during later (i.e. decision-making [11]) stages as opposed to early stages (c.f. [35]).

Finally, Studies 2a and 2b explore whether older adults’ abilities in emotional prosody recognition can be improved through neuro-stimulation of inferior frontal cortex areas using two different neuro-stimulation techniques, namely tDCS and tRNS.

## 2. Study 1: EEG

### 2.1. Methods

All studies (EEG, tDCS, tRNS) and their procedures were approved by the Science and Health Faculty Ethics Subcommittee of the University of Essex (SP1702). We aimed to exceed sample sizes reported in the literature that report ERP group differences (e.g. [39]). All procedures were carried out in accordance to the Declaration of Helsinki, and participants gave written informed consent before participating.

#### 2.1.1. Participants

Sixty individuals were invited to participate through established email lists and newspaper adverts. Of these, data from eighteen participants were not usable and therefore not analysed due to varying reasons (e.g., technical difficulties, broken electrode cap, participant unable to finish experiment). The remaining participants (n = 42) were split into two age-groups (n = 21 each): a younger group (mean_age_ = 21.52, SD_age_ = 2.58; 11 women; 100% university educated) and an older group (mean_age_ = 67.90, SD_age_ = 4.53; 16 women; 12 university educated). All participants reported: (1) English as their native language, (2) right-handedness, (3) having no hearing problems, and (4) having normal or corrected-to-normal vision.

#### 2.1.2. Materials

Stimuli were taken from a previously published inventory [58]. Specifically, we presented twenty-eight pseudo-sentences (e.g. “Barl pranned the cacted yald") that were spoken in different emotions (anger, disgust, fear, happiness, sadness, pleasant surprise, and neutral) by a British actress. Participants were thus presented with 196 pseudo-utterances in total. Pseudo-utterances were used to ensure that participants could not derive emotional meaning from lexical-semantic content, an approach commonly used in emotional prosody research [32, 39, 58] when it is crucial to disentangle the influence of semantic information from prosody processing.

#### 2.1.3. Procedure

Upon entering the lab, participants gave informed consent and provided background information. Participants were then prepared for EEG recordings and were seated in a shielded chamber, at a distance of approximately 100 cm in front of a laptop. Participants were asked to indicate which emotional tone of voice the speaker had used by clicking on one of seven response options displayed on screen. Audio materials were presented via loudspeakers set to a comfortable listening level. The presentation of five practice trials ensured that participants understood the task. Upon successful completion of the practice round, a total of 196 sentences were pseudo-randomly presented via the Superlab software [59] distributed over seven blocks. After each block (twenty-eight trials), participants took a self-determined break, approximately one minute long. For each trial, a fixation cross was presented in the middle of the screen for 250 ms, followed by the presentation of an utterance via speakers. At the offset of the utterance, a response screen was displayed until participants made their choice. Response options were labelled as anger, disgust, fear, happy, surprise, sad, neutral. An interstimulus interval of 1000 ms (blank screen) was included before the next trial began. Participants were encouraged to respond as quickly and accurately as possible. Run time of the experiment was approximately 30 minutes.

#### 2.1.4. ERP recording

We recorded the EEG from 63 Ag-AgCl electrodes mounted on a custom-made cap (‘waveguard’) following the modified extended 10–20 system, using an amplifier by ANT-Neuro. Signals were recorded continuously with a bandpass filter set between DC and 102 Hz and digitized at a sampling rate of 512 Hz. Electrode resistance was kept below 10 kOhm. The left mastoid was used to place the reference electrode, but data were re-referenced offline to averaged mastoids. Bipolar horizontal (positioned to the left and right side of participants’ eyes) and vertical electrooculograms (EOGs; placed below and above the right eye) were recorded for artifact rejection purposes using disposable AmbuBlue Sensor N electro-cardiogram (ECG) electrodes. Data were filtered offline with a cut-off of 30 Hz (using an FIR filter provided by EEP software). Baseline correction was applied. For each channel, the mean of our baseline time window (-200 to 0 ms) was subtracted from the averaged signal. We visually inspected data to remove correctly answered trials containing artifacts and drifts. Automatic EOG artifact rejection (activity above 30.00μ) was applied using the EEProbe software. Approximately 11.5% of data was rejected (range for emotional categories: 10–13%). Artifact data was time-locked to the onset of the stimulus and averaged for 800 ms with a pre-stimulus baseline (200 ms).

#### 2.1.5. ERP data analysis

Electrodes were grouped according to scalp regions of interests (ROI); left frontal: F5, FC3, F1; left central: CP5, C3,CP1; left posterior: P5, PO3, P1; right frontal: F6, FC4, F2; right central: CP6, C4, CP2; right posterior: P6, PO4, P2; and midline: FZ, CZ, PZ. ERP time windows for correctly answered trials were selected based on a combination of visual inspection, past evidence [39] and determining peak latency, see e.g. [60]. Following this approach, the early time window (P2) was set to 180–280 ms and the later time window was set to 280–400 ms.

As only correctly answered trials were included in the ERP analysis, the emotion category of ‘happiness’ had to be excluded from this analysis as participants struggled to recognise this particular emotion. The low recognition rates, paired with additional data loss from the EEG data rejection (e.g. eye movements and muscle artefacts) process, resulted in too few trials that could be averaged to obtain an ERP for this category for the majority of participants. For the same reason, one participant from the elderly group was removed from the EEG analyses.

### 2.2. Results

Firstly, we performed frequentist Analyses of Variance (ANOVA) with Bonferroni post-hoc tests. Where required, p-value for within-subjects comparisons were corrected using the Greenhouse-Geisser approach. We also performed the Bayesian equivalent to obtain the Bayes Factor (BF). BF was calculated using JASP in its default settings for the a priori distribution of the parameters [61, 62]. BF is an index that quantifies the degree of evidence in favour of either the null or the alternative hypothesis. Values of the BF in the range of 3 and 10 provide an index of moderate support for the alternative hypothesis (while values larger than 10 provide stronger support). Conversely, values in the range of 1/3 and 1/10 indicate moderate support for the null hypothesis (and values smaller than 1/10 provide stronger support). BF values in the range of 1/3 and 3 are inconclusive [63].

#### 2.2.1. Behavioural results

Following previous approaches, e.g. [58], an unbiased hit rate (H_u_ score) was calculated to account for response biases before conducting statistical analyses [64]. However, to allow for comparisons across studies, we display the results of average accuracy rates as percentages in tables. Table 1 shows that younger participants were better at identifying emotional prosody than older participants (overall, 75.75% vs 68.81%). For both groups, stimuli expressing anger, sadness and neutral had the best recognition rates, while stimuli expressing happiness had the worst recognition rates. To examine whether emotion recognition differed between the two groups, a 2 (group: younger vs older adults) × 7 (emotion: anger, disgust, fear, happiness, neutral, sadness, pleasant surprise) ANOVA was conducted using the H_u_ scores of each age-group’s average accuracy rate (dependent variable). Omega-squared (ω^2^) was used as an indicator of effect sizes in our ANOVA. Even though effect sizes are context-dependent, an ω^2^ = .010 (i.e. 1% of variance explained) is typically considered a small effect in the literature, an ω^2^ = .059 a medium effect, and ω^2^ = .138 a large effect [65, 66].

**Table 1:** Confusion matrix displaying patterns of average accuracy rates (in %) per emotion category (during the EEG study), grouped by age-group.

Group	Perceived emotion	Intended emotion						
		Anger	Disgust	Fear	Happiness	Neutral	Sadness	Pleasant surprise
Older	Anger	**86.57**	1.70	4.25	1.87	2.21	0	0.34
	Disgust	11.57	**65.99**	3.23	3.06	5.95	1.02	0.34
	Fear	0.68	2.38	**56.97**	2.04	0.34	10.37	0.85
	Happiness	0.34	2.89	4.76	**34.86**	2.38	0.68	28.23
	Neutral	0.51	5.95	7.14	37.93	**84.18**	3.23	1.19
	Sadness	0	1.87	16.67	3.57	3.57	**84.35**	0.34
	Pleasant surprise	0.34	19.22	6.97	16.67	1.36	0.34	**68.71**
Younger	Anger	**92.35**	1.70	0.85	0.51	0.85	0	0.34
	Disgust	4.42	**78.74**	0.34	1.87	3.91	0.34	0.51
	Fear	1.02	1.02	**64.29**	0.68	0.51	11.22	0.17
	Happiness	0.51	3.06	6.63	**52.21**	1.02	0	28.91
	Neutral	0.34	5.10	5.61	32.48	**90.82**	4.93	1.19
	Sadness	0	2.04	18.03	2.04	1.70	**83.16**	0.17
	Pleasant surprise	1.36	8.33	4.25	10.20	1.19	0.34	**68.71**

Note: The percentage accuracy is presented in this table (rather than the H_u_ scores) to allow for comparisons with previously reported findings using percentage accuracy. Unbiased hit rates (H_u_ scores) are available from the supplementary materials. The bold numbers on the diagonals refer to percentage recognition accuracy.

There was a significant main effect of age, F(1, 40) = 7.53, p = .009, ω^2^ = .07, showing that younger participants were significantly better at identifying emotional prosody than older participants. The outcome of the Bayesian analysis was in the same direction albeit inconclusive, BF = 2.53. A significant effect of emotion, F(6, 24) = 65.12, p < .001, ω^2^ = .048, (BF > 100), suggests better recognition for some emotions than others. There was no significant interaction between age and emotion, F(6, 240) = 1.534, p = .198, ω^2^ = .01. The BF = 0.233 provides moderate support for the additive model (i.e. supporting the null hypothesis regarding the absence of the two-way interaction).

**Fig 1:**
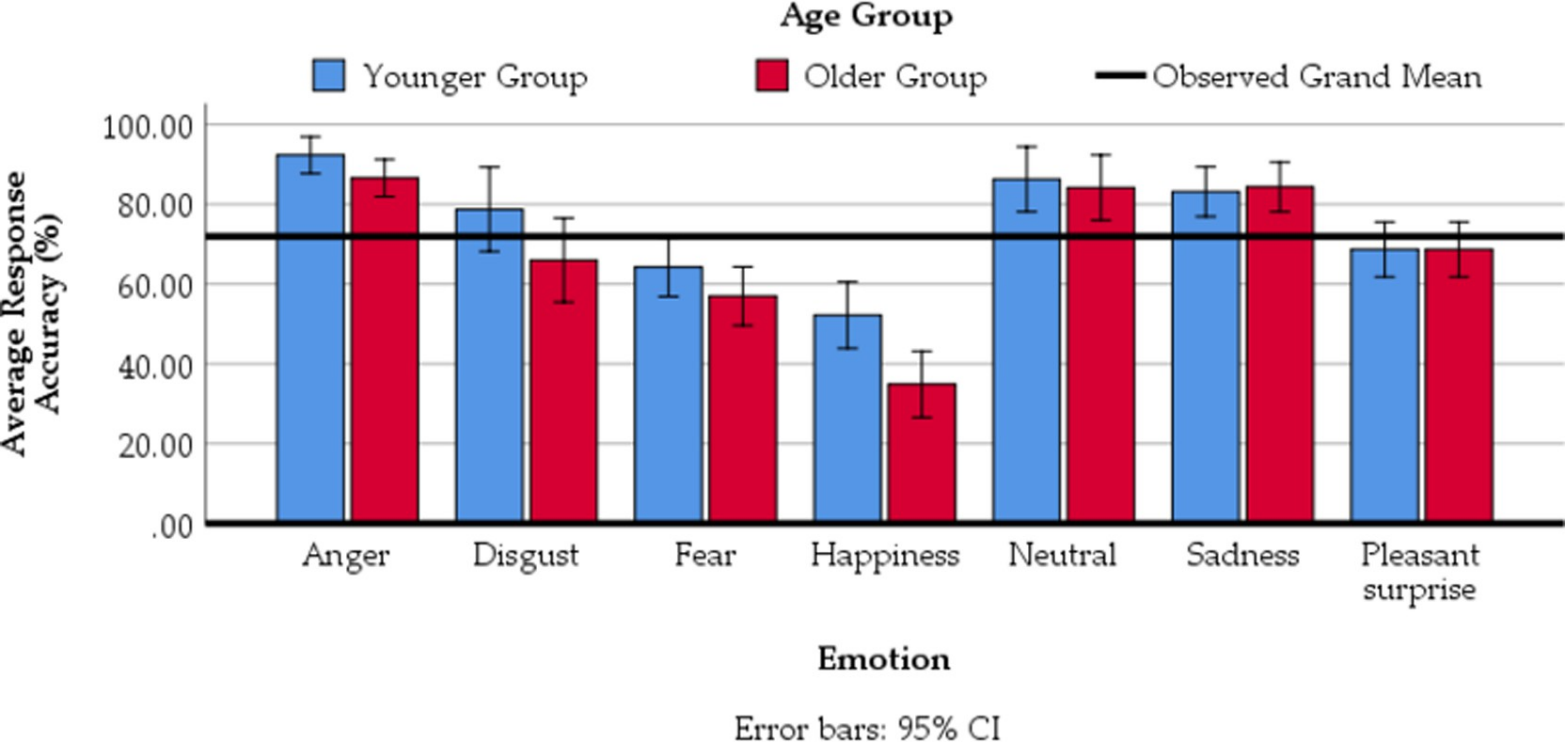
Average accuracy rates (i.e. the number of correctly perceived emotional prosody instances, expressed as a percentage) by age-group.

The main effect of emotion was followed up with Bonferroni post-hoc tests. Results showed significant differences between responses made to angry-sounding stimuli and each of the other emotional categories, ts(41) > 7.43 ps < .001, BFs > 100 (disgust, fear, happiness, neutral, sadness, pleasant surprise). Differences were also found between disgust and fear (t(40) = 3.01, p < .05, BF = 8.05) and disgust and pleasant surprise (t (40) = 3.50, p < .01, BF = 27.1). Response rates to fear differed from happiness and sadness, ts(40) > 6.6, p < .001, BFs > 100. Happiness differed from disgust, neutral, sadness and pleasant surprise, ts(40) > 8.07, p < .001, BFs > 100). Finally, differences were found between sadness and pleasant surprise, t(40) = 4.99, p < .001, BF > 100. For a visualisation of effects see Fig 1.

#### 2.2.2. Discriminant analysis results

Past research (e.g., [36]) suggests that evaluation of how participants use acoustic cues can contribute to our understanding of why groups perform differently in an emotional prosody recognition task. To this aim, we explored if the two groups displayed similar use of acoustic cues by running a discriminant analysis to evaluate whether errors made by participants could be predicted from acoustic cues of stimuli [36]. Acoustic cues (mean fundamental frequency, amplitude, and duration of stimuli; extracted using Praat [67]) of misclassified stimuli were used as the predictor variables while mislabelled emotion served as the dependent variable. Stimuli were grouped according to their most frequent misclassification (older group, n = 185 misclassified stimuli; younger group, n = 178 misclassified stimuli). The discriminant analysis showed that less than half of errors could be predicted by the selected acoustic cues alone (younger group 48.3% vs. older group 36.2%). Looking at success rates by age group and for each emotional category separately, analyses for the older group revealed that the misperceived emotion of anger could not be correctly predicted (0%) by the discriminant functions correlated to the main acoustic cues, while mislabelling stimuli as disgust (21.1%) fear (52.6%), happiness (61.8%), neutral (33.3%), sadness (33.3%) and pleasant surprise (33.3%) led to higher prediction rates. In contrast, for the younger group, mislabelling vocal exemplars as anger was explained by acoustic cues for 62.5% of misclassification. Similar classification results were found for sentences mislabelled as disgust (55.6%), fear (63%) and happy (67.6%). Mislabelling sentences as neutral (34.3%), sad (17.4%) and pleasant surprise (4.8%) were less well explained through acoustic cues. Finally, the discriminant functions were similar for both groups. The first function explained 52.8% (canonical r^2^ = .26) of the variance for older adults and 68% (canonical r^2^ = .43) of the variance in the younger group. It correlated most strongly with mean pitch for both groups. The second function explained 35.6% (canonical r^2^ = .19) of the variance for the older group and 25.4% (canonical r^2^ = .22) for the younger group and correlated with mean amplitude. The third function correlated most strongly with duration and explained 11.6% (canonical r^2^ = .07) of the variance for older and 6.6% (canonical r^2^ = .07) of the variance for the younger group.

#### 2.2.3. ERP results

**Fig 2:**
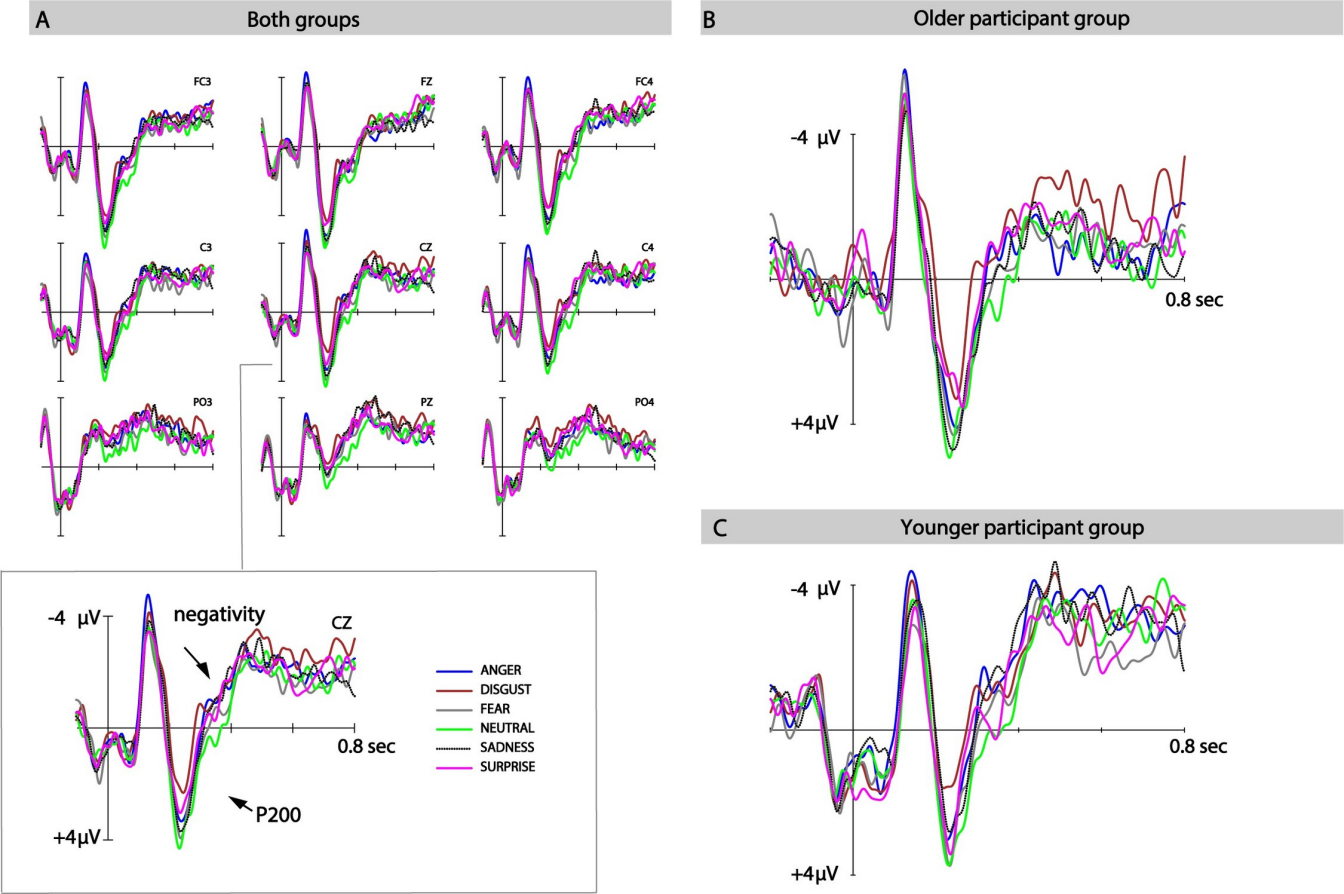
ERP mean amplitude of the younger and older groups per emotion category. Zoomed-in on CZ for closer visual inspection of the data.

Mean ERP amplitudes for the two time-windows of interest (P200, following negative-going brain wave) were analysed using general linear mixed models (GLM) employing a 2 (group: younger vs older adults) × 7 (ROI: left/right frontal, central, parietal; midline electrode sites) × 6 (emotion: anger, disgust, fear, neutral, pleasant surprise and sadness) design. Visualisations of ERPs at selected electrode sites can be found in Fig 2.

*P200*: *180–280 ms*. Analyses revealed a significant main effect of age F(1, 39) = 4.54, p = .05, ω^2^ = 0,08, showing stronger P200 amplitudes for older participants than for younger participants. However the BF = 1.6 is inconclusive regarding age effects. The main effect of emotion was significant, F(5, 195) = 2.76, p < .05, ω^2^ = 0,04, BF > 100, revealing that P200 amplitudes differed between emotion categories. Following past approaches (e.g. [32]), post-hoc tests compared responses of neutral-sounding stimuli to all other emotions. No significant differences were found in the contrast between neutral and anger (t(40) = 1.37, p = .18, BF = 0.4) or neutral and fear (t(40) = .73, p = .47, BF = 0.22), but P200 amplitudes in response to neutral stimuli differed from responses to disgust: t(40) = 3.76, p < .001, BF = 52, pleasant surprise: t(40) = 2.18, p < .05, and sadness: t(40) = 2.2, p < .05. However, the BFs for these comparisons were inconclusive (BF = 1.4 and BF = 1.45, respectively). Crucially, the interaction between age and emotion was not significant (F (5, 195) = .22, p = .913, ω^2^ = 0,0, BF < 1/100) nor was the three-way interaction (F (30, 1170) = .97, p = .51, ω^2^ = 0,0. BF < 1/100).

*Negativity*: *280–400 ms*. Analyses revealed a non-significant effect of age (F(1,40) = 2.13, p = .15, ω^2^ = 0,03, BF = 0.69). The main effect of emotion reached significance F(5, 195) = 2.75, p < .05, ω^2^ = 0,04. Planned post-hoc contrasts between neutral and emotional stimuli revealed no significant differences between neutral and fearful sounding stimuli (t(40) = .98, p = .34, BF = 0.26), but ERP amplitudes in response to neutral stimuli differed from responses to anger, t(40) = 2.7, p = .01, BF = 3.99; disgust, t(40) = 3.14, p < .01, BF = 10.9; pleasant surprise, t(40) = 2.74, p < 0.01, BF = 4.39; and to sadness: t(40) = 3.62, p < 0.01, BF = 36. Visualisation of these effects can be found in Fig 2. Finally, the interaction between age and emotion was not significant (F (5, 195) = 0.29, p = .9, ω^2^ = 0,0, BF < 1/100) nor was the three-way interaction (F (30, 1170) = 1, p = .46, ω^2^ = 0,0, BF < 1/100).

In summary, results revealed no differences between younger and older participants in how emotional voices were responded to in the two ERP time windows of interest.

## 3. Study 2a: tDCS

### 3.1. Methods

To follow up the findings that older adults differ from younger adults in accuracy rates at later decision-making stages, two follow-up studies were conducted using neuro-stimulation techniques. Studies 2a and 2b used tDCS and tRNS, respectively, on the inferior frontal cortex to assess whether electro-stimulation could improve older adults’ ability to correctly identify emotional prosody. Both follow-up studies re-used the experimental task from Study 1.

#### 3.1.1. Participants

A new group of 64 right-handed volunteers were recruited. Four young adult participants withdrew/were excluded during the study, leaving the final total at 30 young adults (mean_age_ = 21.93; 60% women; 15 years on average spent in education), and 30 older adults (mean_age_ = 68.57; 77% women; 15 years on average spent in education). Of the participants who were not included, one withdrew without explanation; the remaining 3 related to adverse reactions to the stimulation (see [68], for further details).

In addition to our standard inclusion criteria (English as native language, no hearing problems, normal to corrected-to-normal vision), participants also completed the transcranial magnetic stimulation Adult Safety Screen (TASS), which included an additional question “Have you ever had an adverse reaction to Transcranial Direct Current Stimulation?” [69]. Participants confirmed that they did not meet any of the contraindications. In addition, they confirmed that they were not taking any medications that affect the central nervous system.

Older participants also completed the Mini-Mental State Examination (MMSE). The MMSE is a frequently used 30-item assessment of cognitive functioning, with a maximum score of 30. Although scores between 24–30 indicate the absence of cognitive impairment, we included only individuals who scored ≥ 26.

#### 3.1.2. tDCS stimulation

A DC-Stimulator Plus (Neurconn, Germany) was used to deliver 2 mA of current for a 30 min duration via two 5 x 7 cm sponge electrodes (NaCl concentration: 100 mM dissolved in distilled water). The electrodes were secured with rubber straps, over F8 (anode) and FP1 (cathode) regions according to the 10–20 system. Electrode location was chosen based on past research [49] and additional motivated by theoretical considerations [35]. The study was double-blinded using the ‘study mode’ function of the stimulator. The active stimulation session began with a 30s ramp-up followed by 30 min of stimulation and 30s ramp-down. During sham, participants received identical 30s ramp-up and–down phases but with 1 min active stimulation in between. The sham stimulation protocol is automatically set by the stimulation device (see [68], for further details).

All participants were told that they would receive active stimulation throughout the study, to enhance blinding efficacy. Comfort of the stimulation was measured at the start and end of stimulation. All participants were administered both active and sham stimulation over 2 sessions, at least 7 days apart. Stimulation condition order was counter-balanced across participants, within each age group. All participant received the F8(anode)/FP1(cathode) montage with F8 being the ‘active’ site and FP1 being the ‘referent’ location.

#### 3.1.3. Behavioural data analysis

To test for performance differences between the two age-groups, we ran a 2 (younger vs older adults) × 2 (tDCS condition: active vs sham) × 7 (emotion: anger, disgust, fear, happiness, sadness, pleasant surprise, neutral) ANOVA. The accuracy rate served as the dependent variable.

### 3.2. Results

Overall, younger participants were again better at identifying emotional prosody from pseudo-utterances than older participants. This holds true for both active (79.18% vs 63.25%) and sham (78.11% vs 64.37%) stimulation. Table 2 shows older adults’ average accuracy rates (%) per stimulation condition (active vs sham).

**Table 2:** Confusion matrix displaying patterns of average response accuracy (in %) per emotion category, per tDCS condition (active, sham), for older adults.

Group	Perceived emotion	Intended emotion						
		Anger	Disgust	Fear	Happiness	Neutral	Sadness	Pleasant surprise
Older (active)	Anger	**85.48**	2.14	4.17	1.79	0.95	0.12	0.71
	Disgust	9.29	**66.07**	4.29	5.48	5.00	0.71	2.26
	Fear	1.90	4.17	**53.69**	2.26	1.19	13.10	1.90
	Happiness	0.71	5.71	8.81	**33.69**	1.19	1.31	36.55
	Neutral	1.90	12.38	13.33	42.86	**84.52**	13.69	9.52
	Sadness	0.36	4.40	13.33	6.90	6.55	**70.95**	0.71
	Pleasant surprise	0.36	5.12	2.38	7.02	0.60	0.12	**48.33**
Older (sham)	Anger	**87.74**	2.86	4.52	2.02	2.02	0.12	0.36
	Disgust	8.45	**65.83**	5.71	4.29	4.40	1.31	3.45
	Fear	1.19	3.21	**51.79**	2.38	0.95	12.02	1.43
	Happiness	0.12	5.83	7.86	**34.29**	2.98	1.43	35.95
	Neutral	1.79	12.38	11.67	42.62	**84.29**	9.17	7.38
	Sadness	0.60	4.17	15.71	6.31	5.36	**75.71**	0.48
	Pleasant surprise	0.12	5.71	2.74	8.10	0	0.24	**50.95**

Note: The bold numbers on the diagonals refer to percentage recognition accuracy.

Results showed a significant main effect of age, with younger participants outperforming older participants, F(1, 58) = 21.96, p < .001, ω^2^ = 0.26, BF>100. There was also a significant main effect of emotion, F(6, 348) = 104.05, p < .001, ω^2^ = 0.62, BF > 100, suggesting that emotion recognition differed between categories. This main effect was informed by a significant interaction between age and emotion, F(6, 348) = 3.07, p = .012, ω^2^ = 0,01, BF > 100. Simple main effects indicated that younger adults outperformed older ones in each emotion, Fs > 8.11, Ps < .006. Crucially, none of the interactions were significant: tDCS by age, F(1,58) = 1.46 , p > .23, BF = 1/5; Emotions by tDCS, F(6, 348) = .94 , p = .46, BF < 1/100 and the three way interaction, F(6, 348) = .56, p = .76, BF = 1/100.

## 4. Study 2b: tRNS

### 4.1. Methods

In a second step, tRNS stimulation was used to determine whether older adults’ abilities for identifying emotional prosody can be improved. To test this, no control group was needed.

#### 4.1.1. Participants

Fifteen right-handed older adults (mean_age_ = 68.13; 53% women; 13 years on average spent in education) took part in this study. The same screening procedures were followed as for the tDCS study. Participants did not take part in the EEG study nor in the tDCS study. Young adults were not included in this study.

#### 4.1.2. tRNS stimulation

A DC-Stimulator Plus (Neurconn, Germany) was again used to deliver 2 mA of tRNS current for a 30 min duration via two 5 x 7 cm sponge electrodes (NaCl concentration: 100 mM dissolved in distilled water). The electrodes were secured with rubber straps, over F8 (anode) and FP1 (cathode) regions according to the 10–20 system. The study was double-blinded using the ‘study mode’ function of the stimulator. The active stimulation session began with a 30s ramp-up followed by 30 min of stimulation and 30s rampdown. The same sham protocol was followed as for the tDCS study.

All participants were told that they would receive active stimulation throughout the study, to enhance blinding efficacy. Comfort of the stimulation was measured at the start and end of stimulation.

Participants were administered active and sham stimulation over 2 sessions, at least 7 days apart. Stimulation condition order was counter-balanced across participants, within each age group. All participant received the F8(anode)/FP1(cathode) montage with F8 being the ‘active’ site and FP1 being the ‘referent’ location.

#### 4.1.3. Design

To examine if neuro-stimulation through tRNS would lead to improvements in older adults’ ability to correctly perceive emotional prosody from speech, accuracy rates were entered into a 2 (tRNS condition: active stimulation vs sham) X 7 (emotion: anger, disgust, fear, happiness, sadness, pleasant surprise, neutral) ANOVA.

### 4.2. Results

#### 4.2.1. Descriptive statistics

Table 3 shows similar recognition rates for older participants during active stimulation (, 55.67%) relative to sham (54.30%).

**Table 3:** Confusion matrix displaying patterns of average response accuracy (in %) per emotion category, per tRNS condition (active, sham), for older adults.

Group	Perceived emotion	Intended emotion						
		Anger	Disgust	Fear	Happiness	Neutral	Sadness	Pleasant surprise
Older (active)	Anger	**79.62**	2.10	3.57	2.31	2.10	0	1.05
	Disgust	15.34	**52.10**	10.08	6.72	4.20	1.89	4.62
	Fear	1.68	5.25	**41.81**	2.73	2.10	14.71	2.94
	Happiness	0.63	10.92	7.98	**25.84**	3.36	2.73	30.67
	Neutral	2.73	13.24	18.28	42.65	**77.94**	15.34	11.34
	Sadness	0	4.41	14.71	7.35	7.77	**63.66**	0.63
	Pleasant surprise	0	11.97	3.57	12.39	2.52	1.68	**48.74**
Older (sham)	Anger	**81.38**	2.04	4.59	1.53	1.79	0	1.28
	Disgust	14.29	**50.26**	6.38	8.16	6.89	2.30	6.38
	Fear	0.77	3.83	**37.76**	4.34	2.04	10.97	2.30
	Happiness	0.26	10.20	9.18	**23.21**	2.55	4.08	32.65
	Neutral	2.30	13.27	20.92	43.88	**75.00**	13.78	8.42
	Sadness	0.77	6.12	16.58	6.63	7.65	**64.03**	0.51
	Pleasant surprise	0.26	14.29	4.59	12.24	4.08	4.85	**48.47**

Note: The bold numbers on the diagonals refer to percentage recognition accuracy.

#### 4.2.2. Average accuracy rates of older adults after tRNS stimulation

There was a significant main effect of emotion, F(6, 84) = 18.64, p < .001, ω^2^ = 0,5, BF > 100. Follow-up tests revealed that the emotion of anger was significantly different from all other emotions tested (ts(13) > 5.71, p < .001). Disgust was significantly different from happiness (t(13) = 4.64, p < .001), and happiness from neutral, sadness and pleasant surprise (ts(13) > 4.9 p < .001). All relevant BF were > 100. There was no significant main effect of tRNS condition (F (1, 14) = 2.21, p = .159, ω^2^ = 0,07, BF = 1/5). There was also no significant interaction of tRNS condition and emotion (F(6, 84) = 0.6, p = .73, ω^2^ = 0,0, BF = 3/100), suggesting that recognition rates could not be improved through tRNS.

## 5. Discussion

The aim of the present investigation was to examine how emotional prosody identification is affected by healthy aging. We examined ERP and behavioural responses to explore different stages of the identification process. We also explored the extent to which older adults’ emotional prosody identification could be improved through the use of tDCS and tRNS. While both ERP components in response to emotional speech were modulated similarly in older and younger participants, older adults showed reduced emotional speech recognition rates. These data suggest that processes linked to emotional-salience detection as well as initial meaning-build-up-related processing are not heavily affected by healthy ageing, while later processing linked to decision-making processes differs across groups. However, attempts to improve older adults’ emotional prosody identification through tDCS and tRNS stimulation were not successful. The implications of these findings are discussed in turn below.

### 5.1. Emotional speech identification unfolding in time

In line with previous findings [8, 11, 20, 25], recognition results confirmed difficulties for aging participants when identifying emotional intentions from voice cues. However, no emotion-specific deficit was observed: older adults were able to detect emotional prosody, but their overall ability to do so effectively consistently seemed to decline with increasing age. Indeed, performance seemed to mirror younger participants’ performance but at a lower success level. For example, while older adults were outperformed by young participants for recognition of anger prosody, they nevertheless recognised many of the stimuli accurately (>80%). Thus, these findings lend additional support to the hypothesis put forward by Ruffman et al. [11], that recognition of basic emotions becomes relatively impaired in older adults regardless of the type of sensory input (i.e. visual, auditory). The question that naturally follows is why older adults perform less well in the identification task.

To address this question, we employed an experimental design that allows to investigate emotional speech perception at different processing stages. Specifically, we first investigated whether older adults detect emotional significance of an auditory stimulus in a similar manner compared to younger participants. Over the past decade, the P200 component has been continuously linked to reflect a neuronal response to emotionally significant acoustic cues (e.g. pitch, loudness, speech rate, voice quality) that listeners use to infer emotional salience. There is evidence that this early emotional salience detection is accompanied by valence and arousal tagging processes, see [57]. Although less well-established, some research also suggests that 200 ms of vocal stimulation are sufficient to activate specific emotional categories [70] rather than just broad valence differentiation. This first emotional differentiation is a crucial sub-step in the recognition process and does not seem to be affected heavily by healthy aging given comparable P200 amplitude modulations between older and younger participants. This is supported by Wood and Kisley [41], who also found no age-related differences in the P200 ERP component, but relying on visual stimuli. This supports the idea that emotional-salience detection occurs similarly between both age-groups regardless of the sensory input (e.g. visual, auditory), which would also be consistent with the findings by Ruffman et al. [11] and work summarised by Kotz and Paulmann [54].

A next step in the recognition process requires close mapping of acoustic and prosodic features onto emotional representations. In the course of emotional prosody unfolding while speakers talk, listeners have to conduct a combinatorial analysis of features available to them; this can include acoustic features, arousal cues, or semantic information. Some past studies have reported a first emotional in-depth processing starting around 300 ms after stimulus onset [32, 71, 72], while the majority of research points to this process being particularly amplified around 400 ms (e.g. [33, 70]). As part of the evidence for an early start of inferring how a speaker feels, an early negative ERP component directly following the P200 has previously been reported [39] when employing an implicit emotional prosody perception task (i.e. not focused on the emotionality of the stimulus). Here, a component similar in morphology and time-course is observed under explicit (i.e. emotionality focused) task instructions. The observation that the component seems to be task focus independent lends support to the idea that it is a mandatory, crucial step to help evaluate emotional-meaning relevant details during emotional speech processing. Interestingly, similar to the P200 component, both age groups show similar ERP modulations at this time point, meaning that both groups show an additional differentiation between emotional and neutral sentences between 280–400 ms after speech onset. This suggests that online processing of emotionally relevant attributes (i.e. information that distinguishes input from neutral stimuli) is not severely impacted by healthy aging at this point. Based on these combined EEG results, we argue that older and younger participants recruit a similar neural network during early stages of emotional speech comprehension. Moreover, looking at brain-based models of emotional prosody processing [35, 54], they postulate involvement of auditory cortices and right anterior superior temporal sulcus as well as superior temporal gyrus in these processing steps. Later, decision-making stages as tested with our recognition task are argued to be modulated by inferior frontal and orbito-frontal cortices, areas prone to brain atrophy in healthy aging [11]. It should be noted that our groups were relatively small (n = 21) and it could be argued that sample size would have had to be increased to report significant ERP group differences. We believe that lack of power is not the underlying cause for our null-result finding, however, as past research reporting ERP group differences for neural responses [e.g., the LPP in 41; ERPs following the P200 in [73, 74]] have tested similar sample sizes. Moreover, here we report a lack of group differences for two types of statistical analyses and ERP plots show large similarities across groups.

We only report reduced emotional speech recognition in healthy aging compared to younger participants in the explicit identification task. The finding then lends support to the notion that ageing listeners exhibit difficulties with the explicit evaluation of emotional attributes that require a specific output behaviour (e.g. button-press that indicates the identification of the stimulus). Crucially, no emotion-specific deficit was observed, thereby directly providing evidence against the so called ‘positivity effect’ which postulates that older adults pay more attention to positive stimuli. If true, we should have observed better recognition for stimuli expressing happiness or pleasant surprise; however, here, we find that angry stimuli were recognised best and happy and pleasantly surprised sounding stimuli were more difficult to recognise. Analyses were conducted taking into account response biases; again, we failed to observe that response bias towards positive stimuli occurred. We can only speculate why the ‘positivity-effect’ was not confirmed here, but Kalenzaga et al. [75] have argued that task instructions might modulate these types of effects. This can be explored in future studies. The present findings simply show no evidence for enhanced attention or recognition to positive stimuli. To test, however, if errors made could be predicted by acoustic cues, additional exploratory discriminant analyses were conducted similar to earlier reports in [8]. The next section will look at these findings in more detail.

### 5.2. Acoustic cue use—differences between age-groups?

Past evidence showed age-related differences in emotional speech recognition and linked those finding to differences in acoustic cue usage between younger and older listeners. Specifically, Paulmann et al. [8] report that acoustic cues could better predict what kind of error patterns younger as opposed to older participants made. They speculated that older adults might experience difficulties using the acoustic input successfully when identifying emotional intentions. In the present study, ERP results suggest comparable early auditory processing for both groups, implying that acoustic cue perception per se is not impaired in older listeners. To infer if difficulties in identifying emotional speech stimuli could be linked back to older adults relying more heavily on specific cues, we ran a canonical discriminant analysis which used mean pitch, loudness and duration of mis-classified stimuli to predict responses. Prediction accuracy is below 50% for both groups, but the choice of some emotional categories seem to be predicted more easily than others. For example, none of the stimuli misclassified as angry could be linked back to similarities in acoustic cues when looking at results from *older* adults; however, 62.5% of stimuli that were attributed as ‘angry’ by *young* participants could be explained by the model. This suggests that younger adults’ errors were based more strongly on paying attention to acoustic cues while older adults showed more variability in the way they used cues.

Overall, the lack of clear success in predicting errors through speaker’s vocal cues might be taken as evidence to support the idea that pitch and loudness alone are not as crucial when detecting emotions and that acoustic correlates of voice quality [16, 76], which are not analysed here, may be contributing significant clues as to which emotion the speaker is expressing. One critical difference between acoustic features has been tied back to how much they are affected by physiological changes that influence how emotions are produced. For example, voice quality features (e.g. HNR, proportion of energy present in specific frequency ranges) might be more prone to physiological or hormonal influences than pitch or loudness production [16, 76]. Alternatively, pitch and loudness might help listeners to better differentiate the dimension of valence and/or arousal, but are less beneficial when having to identify specific emotion categories [16, 76, 77]. Finally, it should be highlighted that differences in stimuli properties can contribute to mixed findings. For instance, in our studies, a single actress expressed all stimuli, while in other studies different speakers were used (e.g. gender and/or age differences), which might have evoked different perceptions of familiarity, which could in turn influence listeners’ emotion perception differently [16].

### 5.3. Average accuracy rate after neuro-stimulation

Findings from Study 1 are consistent with evidence from previous studies showing that older adults are worse at perceiving emotion prosody from speech than younger adults. Study 1 also highlighted that processing differences seem to occur at later decision-making stages rather than during early salience or emotional meaning evaluation. Combined, results indirectly support the hypothesis that naturally occurring brain atrophy contributes to emotional identification differences.

To test if neuro-stimulation of frontal cortex would improve older adults’ ability to correctly perceive emotional prosody from utterances, we employed tDCS stimulation at F8 electrode site, attempting to increase neuronal activity needed for emotional decision-making. Contrary to expectations, results for each group revealed no significant differences between active and sham stimulation. In both conditions, younger participants outperformed older participants similar to Study 1.

Although older adults’ ability to perceive emotional prosody from speech was not improved through tDCS, this result may partly be explained by the chosen time-windows during the task; different stimulation times or duration might offer a different effect (c.f. Hebbian theory [78] for further details). Contradicting findings by Francois-Nienaber et al. [53], which suggested a reduction in the perceived emotions of anger and fear, might similarly be due to differences in methodology, as they had used a T-RES (Test of Rating of Emotions in Speech) speech emotion rating paradigm, with special focus on lexical content. Moreover, it is also important to note that the study by Francois-Nienaber et al. [53] involved younger adult participants, which makes no guarantees for any generalisation of their findings towards an older population.

Additional studies by Boggio et al. [79] and Peña-Gómez et al. [80] revealed that anodal-tDCS on the left dorsolateral prefrontal cortex (F3 position) was able to modulate and reduce the negative emotions perceived by participants during a picture task. Peña-Gómez et al. [80], who had 1mA applied for 20 minutes for anodal-tDCS and a cathode positioned on C4, also highlighted the fact that personality variables could be an important aspect to consider for future studies, since they observed that participants higher on the introversion dimension were more permeable to the effects of tDCS than extroverts. Contrary to these studies, we had positioned anodal on F8 (2mA applied for 30 minutes; cathode positioned on FP1). Focus on the left dorsolateral prefrontal cortex at the electrode positions highlighted by Peña-Gómez et al. could be something to take into account for the future.

As part of our neuro-stimulation studies, we also used tRNS on the frontal cortex areas to assess whether older adults’ ability to correctly identify emotional prosody can be improved. To the best of our knowledge, this is the first study to investigate the difference between tRNS active and sham conditions for each basic emotion from uttered pseudo-sentences applied on older adults. Contrary to Yang and Banissy [49] and Penton et al. [50], our findings revealed no improvements in older adults’ ability to correctly discriminate emotions from prosody with tRNS stimulation, regardless of emotion. As with tDCS, this may reflect a genuine lack of effect, or it may relate to differences between the two studies, such as the use of different current strength or the different tasks that were utilised. Both Yang and Banissy and Penton et al. applied high frequency tRNS on the inferior frontal cortex (electrodes positioned on F7 and F8) with current strength of 1mA applied for 20 minutes. Others have also pointed out that the inferior frontal cortex is more sensitive towards anger than positive emotions [81], which would be an interesting aspect to explore in future studies.

### 5.4. Concluding remarks

The findings of the present study provide additional support to a growing body of evidence outlining differences between younger and older adults with respect to emotion processing. Specifically, when looking at the ability to identify emotions from voice cues, older adults’ performance is reduced compared to younger adults (e.g. [8, 11]). This difficulty cannot be linked to age-related differences in early processing steps (i.e. extraction of acoustic cues, and emotional salience detection measured from the P200 ERP component) nor through age-related differences in first in-depth emotional meaning evaluation (i.e. negativity). Rather, following suggestions from multi-step processing models of emotional speech [35, 54], the findings of the present study suggest that decreased accuracy rates in older adults might be due to difficulties at decision-making stages (i.e. explicitly labelling an emotion). While the present investigation failed to induce improvements in emotional speech recognition with two different neuro-stimulation techniques, tDCS and tRNS, this should not be taken as an indicator that these techniques could not be used to help older adults improve their ability to correctly perceive emotional prosody. Rather, methodological changes (e.g. stimulation voltage, duration, time-point or electrode-site location) should be tested in future studies to help further our understanding around emotional communication in healthy ageing. Indeed, future studies could improve on the current methodology by more closely monitoring the influence of cognitive function, socio-economic status or personality information to help tease apart which other variables might contribute to reported group differences.

## Supporting information

S1 Data (XLSX)
